# Fabrication of Laser-Induced Graphene/MoS_2_/PVA@Melamine Evaporators for Rapid Solar Steam Generation

**DOI:** 10.3390/polym18212778

**Published:** 2026-11-07

**Authors:** Entesar A. Ganash, Salihah A. Al-zahrani, Hala A. Al-Jawhari, Reem M. Altuwirqi

**Affiliations:** Physics Department, Faculty of Science, King Abdulaziz University, Jeddah 21589, Saudi Arabia; salihahalzahrani1@gmail.com (S.A.A.-z.); hala.aljawhari@gmail.com (H.A.A.-J.); raltuwirqi@kau.edu.sa (R.M.A.)

**Keywords:** solar-steam generation, desalination, evaporation rate, laser-induced graphene, wettability, photothermal material, MoS_2_, melamine foam, Janus configuration

## Abstract

The increasing global water scarcity, driven by population growth, industrialization, and climate change, has created an urgent need for sustainable and efficient water desalination. This study focuses on developing a solar-driven steam generation (SSG) desalination system that uses photothermal materials (PTMs), such as molybdenum disulfide (MoS_2_) and graphene produced via laser-induced graphene (LIG), to enhance evaporation efficiency. The system was prepared by coating melamine foam (MF), used as the base, with polyvinyl alcohol (PVA) at varying concentrations (0.6 wt%–1.4 wt%), with the 1 wt% concentration showing the highest evaporation rate. MoS_2_ (1 wt%) was incorporated to improve light absorption. The final step involved graphitization and surface modification via the LIG technique, using a 450 ± 5 nm visible laser to induce structural changes. The effects of laser power and scribing density (Lines-Per-Inch, LPI) on the evaporation rate and efficiency were investigated. The optimal configuration, consisting of 1 wt% PVA, 1 wt% MoS_2_, 0.5 W laser power, and 300 LPI, significantly enhanced light absorption to 77%, compared with 65% in the baseline sample. This Janus configuration achieved an evaporation rate of 1.925 kg m^−2^ h^−2^ in saline water (3.5% sodium chloride (NaCl)) under 1 kW/m^2^ solar irradiance, corresponding to an apparent evaporation efficiency of ~98%. The integration of material engineering improves the SSG system’s performance. The system demonstrated excellent stability over 10 cycles in a saline environment, with no significant performance degradation. Using the combined advantages of PVA, MoS_2_ and LIG offers a scalable, cost-effective, and energy-efficient approach, making it a promising system for sustainable desalination and the water scarcity crisis.

## 1. Introduction

Global water scarcity is a growing challenge driven by population growth and economic development. By 2050, nearly four billion people are estimated to face freshwater shortages [1]. This has created an urgent need for efficient desalination technologies [2]. Researchers have explored several desalination methods, including thermal distillation [3], reverse osmosis [4], membrane filtration [5], and electrodialysis [6]. Among these, thermal distillation can generate steam from both natural and contaminated water sources but consumes large amounts of energy, resulting in energy waste and increased pollution [7]. Moreover, conventional solar evaporators have low solar-to-thermal conversion efficiencies (30–45%) because of poor light absorption and significant heat losses, limiting their practicality for large-scale clean water generation [1]. In response, researchers have developed solar steam generation (SSG) systems using photothermal materials (PTMs) [7]. These materials, such as carbon-based substances, plasmonic metals, and semiconductors, absorb sunlight and convert it into thermal energy. This heat is retained in the PTM layer by adding insulators like polystyrene (PS) or polyethylene (PE) foam to reduce heat loss, resulting in higher evaporation rates [8].

Some studies examined solar-driven water evaporation. For example, researchers developed carbon nanoarchitectures by depositing hydrophilic, nitrogen-doped free-standing graphene nanopetals (N-fGPs) on the hydrophobic surface of self-assembled graphene foam (sGF) using plasma-enhanced chemical vapor deposition (PECVD). The N-fGN/sGF construct achieved an evaporation efficiency of 88.6% and a 1.27 kg m^−22^ h^−1^ average evaporation rate over 12 h under 1 sun [9]. In 2026, Wang et al. [10] extensively reviewed inorganic chemical reduction methods to prepare titanium dioxide (TiO_2−x_) and its applications in solar-driven interfacial water evaporation. In addition, Li and coworkers fabricated a novel SSG device [11] which consists of polydopamine nanoparticles (PDA NPs), polyvinyl alcohol (PVA), and polytetrafluoroethylene (PTFE). Its evaporation rate was 2.01 kg m^−2^ h^−1^ under 1 sun.

Molybdenum disulfide (MoS_2_), a layered semiconductor [8], has attracted interest for its exceptional light absorption across both the near-infrared (NIR) and visible wavelengths. The variable bandgap of MoS_2_ (1.3–1.9 eV) enables effective photothermal conversion [12]. It has excellent thermal properties and salt rejection [8]. It is considered an efficient photothermal solar absorber [12]. Because of these properties, MoS_2_ is considered a substitute PTM for water evaporation [13]. Evaporation rates exceeding 1.2 kg m^−2^ h^−2^ have been achieved under a solar intensity of 1 kW/m^2^ (1 sun) by integrating MoS_2_ with various substrates, including carbon-based materials, such as bio-carbon foam and polyurethane sponges [8]. Carbon-based materials, due to their low cost, excellent thermal conductivity, and high energy conversion efficiency, are ideal for use in MoS_2_ composites for SSG [12].

Among carbon-based materials, graphene is notable for its extensive light absorption, though its production remains challenging. In 2014, laser-induced graphene (LIG) was introduced [14]. It is a scalable, solvent-free method for producing graphene via laser scribing. LIG is a porous, graphene-like carbon formed when a laser converts a carbon-containing polymer surface in a single, binder-free step. This technique offers controllable structures and properties, as well as adaptability to various raw materials, making it a promising method for producing graphene materials [15]. For solar evaporation, LIG offers several advantages. First, it provides broadband solar absorption and minimizes energy loss, improving the evaporation region and rate [16,17]. Second, its 3D porous interconnected surface structure [17] supports vapor escape. Third, laser power and scribing density can tune its morphology and surface wettability, allowing the balance between water supply and heat localization to be set by a processing parameter [18]. Fourth, direct laser writing is patternable and scalable. Lastly, LIG combined with PVA improves the structural stability and performance of the resulting structure [17,19].

However, a major challenge in solar-driven desalination is the accumulation of salt crystals on an evaporator surface. These crystals block water and steam transport, reflect sunlight, and reduce energy efficiency [20]. Janus-structured evaporators, which feature a hydrophobic upper layer and a hydrophilic bottom layer, have been developed to mitigate salt precipitation. Nonetheless, these structures face issues such as hindered water supply to the heating region due to overly hydrophobic surfaces and loss of hydrophobicity over time from photochemical oxidation, which reduces salt-rejection ability. To ensure long-term efficiency, the upper surface must balance hydrophobicity and oxidation resistance [21].

This work aims to develop a Janus evaporator with high evaporation efficiency and stable hydrophobic properties. To this end, materials engineering was integrated with the LIG technique. Here, a low-cost, commercially available melamine foam (MF) was used. It is inherently hydrophilic and characterized by superior water transport and thermal insulation; moreover, it has a high evaporation surface area [22]. Next, a dip-coating technique was used to coat the MF surface with PVA, a water-soluble polymer distinguished by its chemical stability, high hydrophilicity, and film-forming ability [23]. MoS_2_ was also incorporated into the PVA layer (abbreviated as MoS_2_/PVA@MF) as a cost-effective light absorber to enhance photothermal conversion efficiency. To further enhance photothermal performance and adjust the surface wettability, the LIG technique was applied to the resulting MoS_2_/PVA@MF composite. The LIG process was applied using a simple, compact, and inexpensive laser system with a visible blue wavelength.

Additionally, this work examined how laser parameters, particularly laser power and scribing density (lines per inch, LPI), tuned the structure’s morphology and its effect on the evaporation rate. By adjusting these parameters, the evaporator surface exhibited moderate hydrophobicity, which optimized heat absorption and retention and consequently enhanced the evaporation rate. Meanwhile, the lower section of the foam retained its hydrophilic, open-cell structure, contributing to the Janus configuration by facilitating continuous water transport while providing effective thermal insulation. This combination of surface wettability, efficient water supply, and thermal management makes the system well suited for solar desalination applications.

## 2. Materials and Methods

MF and PS were purchased locally; PVA (Techno Pharmchem, Bahadurgarh, India) and high-purity MoS_2_ powder (Ossila Ltd., Sheffield, UK) were used to prepare four solar steam evaporators. A diode laser (s30 Pro, SCULPFUN, Shenzhen, Guangdong, China) with a 450 ± 5 nm wavelength, a maximum power of 10 W, and a fixed focus with a spot size 0.06 × 0.08 mm was used for the LIG process. The laser parameters were controlled using LightBurn software (v2.0), including laser power (W), scanning speed (mm/min), and scribing density (LPI ≡ 1/line spacing), to investigate their influence on evaporation. The preparation of PVA and MoS_2_/PVA coatings is explained in detail below, along with LIG fabrication and the SSG experiment.

### 2.1. Preparation of PVA Coating

In this procedure, Janus evaporators were constructed using an MF coated with PVA. Preparation began by dissolving 1 g of PVA in 100 mL of distilled water (DI). The solution was stirred magnetically while heated to 80 °C for 2 h. After preparing the PVA solution, the top portion of the MF, measuring 2 cm × 2 cm × 2 cm, was immersed in the solution, coating only one surface to one-third its thickness. The coated foam was then dried in an oven at 100 °C for 4 h, yielding a PVA/MF composite foam (referred to as FP). This process was repeated with PVA concentrations of 0.6 wt%, 0.8 wt%, 1.0 wt%, 1.2 wt%, and 1.4 wt%, producing composite foams referred to as FP-0.6, FP-0.8, FP-1.0, FP-1.2, and FP-1.4, respectively. The preparation method is illustrated in Figure S1a.

### 2.2. Preparation of MoS_2_/PVA Coating

The MoS_2_/PVA@MF composite foam (named FPM) was prepared by dissolving 2 wt% PVA in DI water under magnetic stirring at 80 °C for 2 h until fully dissolved. Separately, 2 wt% MoS_2_ was dispersed in DI water and stirred magnetically at room temperature for 2 h. The two solutions were then combined to achieve final concentrations of 1 wt% for both PVA and MoS_2_, and the mixture was homogenized by ultrasonic treatment for 2 h to achieve uniform dispersion. The top surface of a 4 cm^2^ square piece of MF was immersed in the prepared solution to a depth of one-third of its thickness to ensure uniform coating. The coated foam was then dried in an oven at 100 °C for 4 h to remove residual moisture. The preparation method is illustrated in Figure S1b.

### 2.3. LIG Fabrication on FPM Evaporator

**Figure 1:**
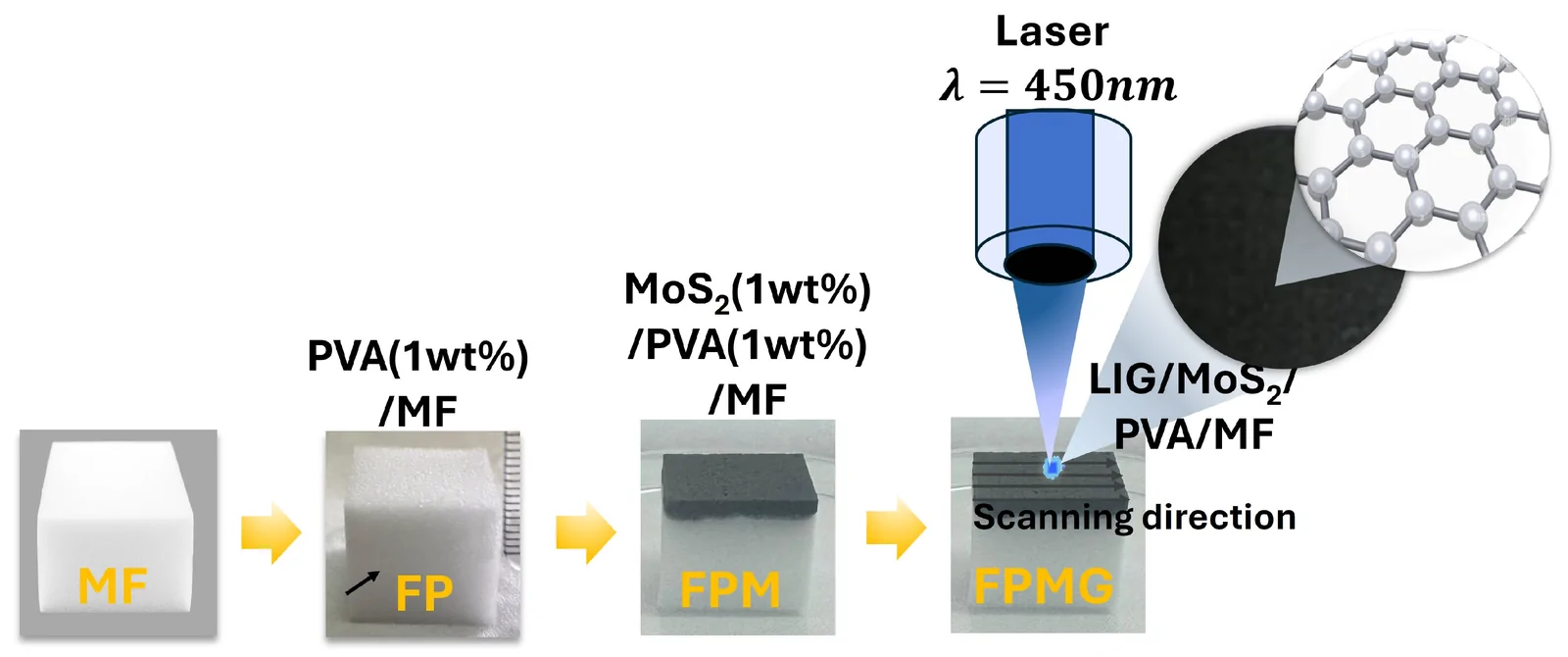
Schematic illustration of the fabrication process for generating the FPMG sample using the LIG method with a 450 nm wavelength. The black arrow in the FP sample indicates the PVA layer.

LIG was directly fabricated on the FPM evaporator using the Sculpfun S30 Pro laser engraving machine to obtain the FPMG sample. A schematic illustration of the fabrication process for generating the FPMG sample is shown in Figure 1. Four laser power levels (5%, 10%, 20%, 30%) were tested relative to the maximum power of 10 W (corresponding to 0.5, 1, 2, 3 W, respectively). The scanning speed was set to 4000 mm/min, and the scribing density was set to 50 LPI as the minimum value. The distance between the laser and the foam was adjusted so that the top surface was at the focal point of the laser beam. The resulting samples were designated FPMG-W, where W denotes the optimal laser power, and their performance was evaluated by the evaporation rate. The sample with the optimal laser power, which showed the highest evaporation rate, was selected for further study of the effect of scribing density. The impact of scribing density on the evaporation rate was tested by selecting three values (50, 100, 300 LPI). The samples were designated FPMG-W-LPI, where LPI indicates the scribing density.

### 2.4. SSG Experiment

To assess water evaporation performance, a system was developed, shown in Figure S1c, consisting of a 100 mL glass beaker filled with 3.5% saline water to replicate seawater salinity. A Janus-type solar evaporator, featuring an upper sunlight-absorbing layer and a thermally insulating, porous lower layer, was mounted in a PS frame and floated on the water’s surface due to its porous structure and density similar to water [24]. A solar simulator (MAX-303, Asahi Spectra, Tokyo, Japan) with a 300 W xenon lamp simulated sunlight at 1 kW/m^2^, and the irradiance was verified using Newport 1918-R optical power meter with 918D-UV-OD3R Photodetector (Newport Corporation, Irvine, CA, USA). Water evaporation was measured using a high-precision electronic balance (KERN & Sohn GmbH, Balingen, Germany) with 0.01 g accuracy, while the temperatures of the PTM and water were monitored with an Infrared (IR) or thermal imaging camera (UTi165A, UNI-T, Dongguan, Guangdong, China). Each test lasted 60 min, with weight measurements recorded every 10 min. All experiments were repeated three times to ensure accuracy.

The rate of water evaporation (v) is given by [25] and determined using Equation (1)

(1) $$v=\frac{\Delta m}{S\times t}$$

where Δ*m* represents the change in mass during water evaporation (kg), *S* denotes the area of the solar absorber (m^2^), and *t* represents the irradiation time (h). The net evaporation rate of water is calculated by subtracting the baseline evaporation in the dark ($v_{dark}$) from the evaporation rate under solar irradiation (v), when performed under the same ambient conditions. This isolates the contribution of solar irradiation in the evaporation process.

The water evaporation efficiency (*η*) due to solar radiation, taking into account the dark evaporation rate $v_{dark}$, is then calculated using Equation (2)

(2) $$\eta =\frac{(v_{light\;}-{\;v}_{dark})\;h_{LV}}{C_{opt\;}q_{in}}$$

$q_{in}$ refers to the normal-incident solar irradiation value of 1 kW/m^2^, and $C_{opt}$ indicates the optical concentration factor, the multiple of 1 kW/m^2^ (≈1 for no concentrator) [25]. $h_{LV}=c\left(T-T_{0}\right)+h_{v}$ denotes the total enthalpy of both sensible heat and latent heat, $h_{v}$ (phase change) for water, where *c* is the specific heat capacity of seawater (4.0 kJ kg^−1^ K^−1^), *T* is the membrane temperature, $T_{0}$ is its initial water temperature, and $h_{v}=2398\;kJ/kg$ at *T* = 47 °C as given by the National Institute of Standards and Technology (NIST) steam tables [26]. For more clarification, see (Note S1) in the Supplementary Information.

### 2.5. Measurement and Characterization

The evaporators’ surface morphologies were examined using field emission scanning electron microscopy (FESEM) (JSM-7600F, JEOL Ltd., Tokyo, Japan). To investigate the PTM’s absorption, reflectance spectroscopy was performed using HR4000 Fiber Optic Spectrometer (Ocean Optics Inc., Dunedin, FL, USA), a halogen light source (Thunder Optics, Padova, Italy), and an integrating sphere (Thorlabs Inc., Newton, NJ, USA). Raman spectroscopy (DXR, Thermo Fisher Scientific, Waltham, MA, USA) was used to investigate the chemical structures of the samples under study. A solid-state laser at 532 nm was used for excitation. It has a 100× lens magnification. Lastly, the contact angle was measured using the sessile drop method by placing a 5 µL droplet of DI water on the foam surface, and images were captured with a digital camera and analyzed using ImageJ software (v1.52).

## 3. Results and Discussion

### 3.1. PVA Concentration Optimization

**Figure 2:**
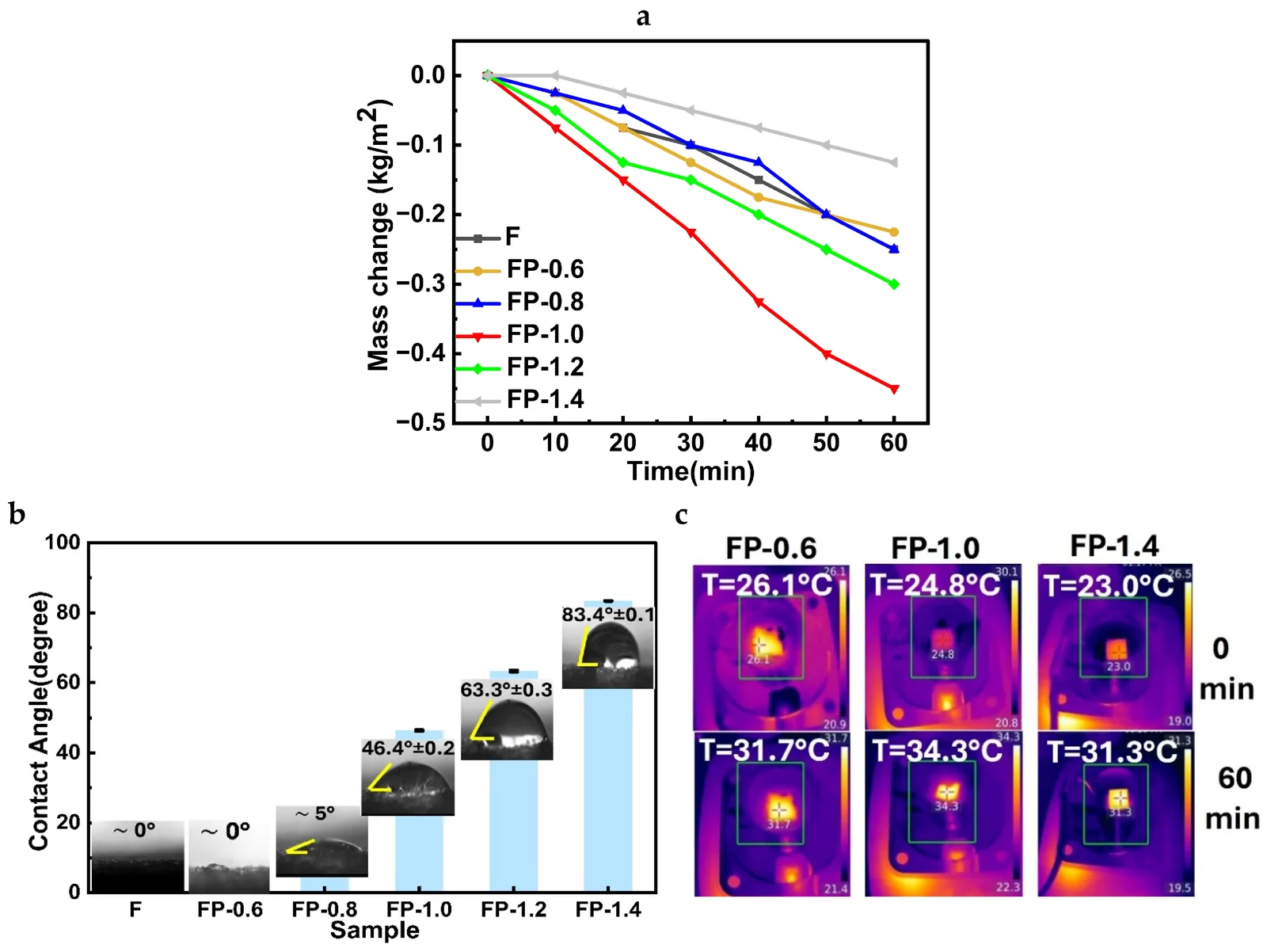
(**a**) The mass change and (**b**) the contact angle measurements of the F sample and the Janus foams coated with various PVA concentrations over time. (**c**) IR images of Janus foams coated with various PVA ratios at 0 min and after 60 min under a one-sun irradiation intensity.

The effect of PVA concentration on the evaporation rate was investigated by coating the MF surface with PVA at varying concentrations. FP-1.0 (1 wt% PVA) achieved the best performance with the highest evaporation rate of 0.45 kg m^−2^ h^−1^, as shown in Figure 2a. To study the probable causes for such performance, the surface wettability and surface temperature were investigated at each PVA concentration. For the FP-1.0 sample, the static water droplet contact angle was ~46.4° (Figure 2b), indicating moderate hydrophilicity that allowed sufficient water distribution across the photothermal surface, which reached 34.3 °C (Figure 2c). This ensured a continuous water supply to the heating region without excessive spreading or heat dissipation, issues observed in highly hydrophilic samples such as the untreated MF (F sample), FP-0.6, and FP-0.8, which have contact angles of ~0°, ~0°, and ~5°, respectively, and exhibited reduced thermal efficiency even with almost a similar surface temperature. Conversely, higher PVA concentrations produced less hydrophilic surfaces with higher contact angles, as observed in FP-1.2 (~63.3°) and FP-1.4 (~83.4°). This reduced hydrophilicity decreased the water supply to the heating region, limiting interaction between water and the photothermal surface. Although PVA is generally hydrophilic, at high concentrations it forms a denser surface layer that increases the contact angle and restricts water flow, thereby negatively affecting evaporation performance. As shown in Figure 2c, the PTM surface temperature after 60 min under one-sun solar irradiation did not significantly depend on the PVA concentration; hence, the surface wettability may be considered the key parameter influencing the evaporation rates. The moderate contact angle (~46.4) and a moderate surface temperature (~34.3 °C) of the FP-1.0 sample enabled the formation of a thin, stable water film and ensured efficient interfacial heat transfer without excessive heat loss, reflecting a balanced thermal response between absorption and dissipation. Therefore, the FP-1.0 sample, with its intermediate PVA concentration and balanced surface properties, provides the optimal combination of wettability and temperature, making it the most likely to exhibit the highest evaporation rate. Therefore, the FP-1.0 sample was chosen for further study.

### 3.2. Effect of MoS_2_ on the Performance of Solar Evaporators

**Figure 3:**
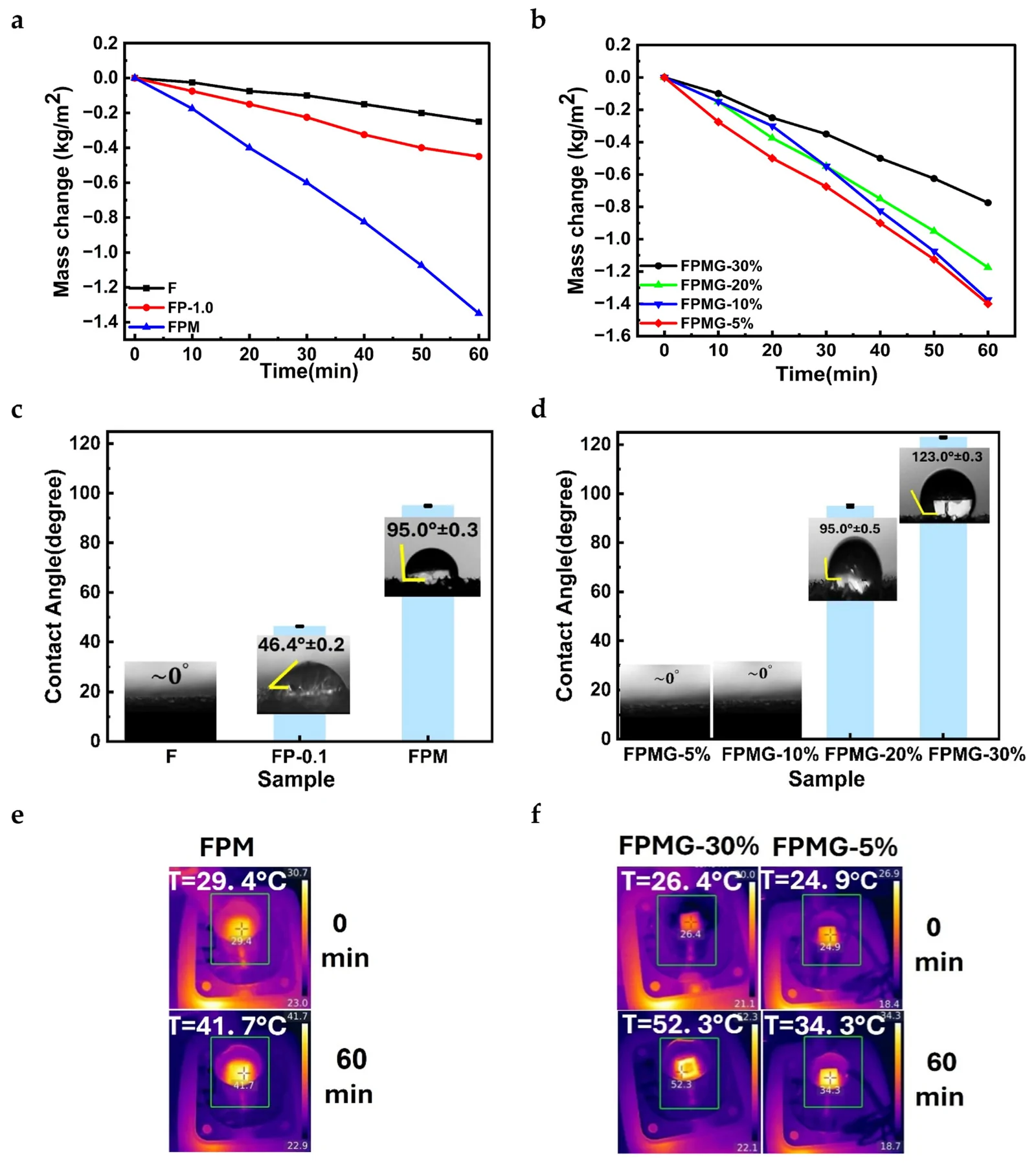
(**a**,**b**) The mass change in FPM and FPMG samples prepared with varying laser power levels (5%, 10%, 20%, and 30%). (**c**,**d**) Contact angle measurements of these samples at the same power levels. (**e**,**f**) IR images of the samples subjected to different laser power levels taken at 0 min and after 60 min, under an irradiation intensity of one sun.

As mentioned above, adding MoS_2_ led to the sample being renamed FPM. The addition of MoS_2_ (1 wt%) significantly increased the evaporation rate to 1.35 kg m^−2^ h^−1^, (Figure 3a) and raised surface temperature to 41.7 °C, (Figure 3c). The contact angle increased to 95°, as shown in Figure 3e, compared to 46.4° at the same PVA concentration (1 wt%) with a surface temperature of 34.3 °C. This enhancement can be attributed to MoS_2_’s synergistic effects on both thermal properties and water-surface interaction. MoS_2_’s broad-spectrum light absorption enhances the conversion of thermal radiation into heat [12], thereby increasing the surface temperature and providing more energy for evaporation. Additionally, the increased contact angle reduced surface wettability, preventing excessive water penetration and optimizing water availability at the evaporation layer. MoS_2_’s high thermal conductivity ensures uniform heat distribution, reducing thermal gradients and maintaining a steady evaporation rate. Collectively, these factors enhanced light absorption, increased surface temperature, and reduced wettability. Hence, they contributed to the significant improvement in the evaporation rate, highlighting the potential of MoS_2_ as a performance-enhancing material for thermal evaporation systems.

### 3.3. Laser Parameters Optimization

After applying a laser to the FPM sample, it was renamed FPMG, as stated earlier. Further parameter investigations were also required. This work focused on two parameters: laser power and scribing density.

### 3.4. Effect of Power

To study the effect of laser power, all other parameters were held constant. Increasing the laser power from 5% to 10% did not significantly change the measured evaporation rate (1.40 kg m^−2^ h^−1^ against 1.35 kg m^−2^ h^−1^ for FPM), as can be seen from Figure 3a,b. However, as power increased to 20% and 30%, the evaporation rates were reduced to ~1.2 and 0.8 kg m^−2^ h^−1^, respectively (Figure 3b). Another aspect is the contact angle, which varied non-monotonically from a hydrophilic 0° for FPMG-5% to a hydrophobic 123° for FPMG-30% (Figure 3d). The sharp transition from a hydrophilic to a hydrophobic surface is attributed to the chemical threshold in laser conversion. At low laser power, the hydrophilic PVA hydroxyl groups are retained. However, above a threshold laser power, the graphitization exposes the hydrophobic sp^2^ carbon and burns off the hydrophilic groups. At elevated laser powers, excessive energy absorption can induce significant structural and chemical changes in the material, such as total surface carbonization, increased roughness, and degradation of functional components, which adversely affect the material’s thermal and wetting properties [27]. As laser power increases, the pristine graphene content in the MoS_2_ film increases accordingly, enhancing hydrophobicity because carbon lacks the polar bonds needed to attract water. This, in turn, improves the composite’s water-repellent properties. The decline in evaporation rates at higher laser power may be attributed to damage or over-carbonization of the PVA/MoS_2_ layer and/or a reduced water supply to the hydrophobic surface. Another aspect to investigate is the surface temperature. For instance, as shown in Figure 3d,f, at 30% laser power, the surface temperature reached 52 °C, while the contact angle reached 123°, making the surface highly hydrophobic and reducing the effective evaporation area and, consequently, the evaporation rate. In contrast, at 5% laser power, the surface temperature was only 34 °C; however, the contact angle was 0°, indicating a super-hydrophilic surface that enhanced water spreading and was seen to improve the evaporation rate. This indicates that surface temperature alone is not the key parameter for increasing evaporation rates. A balance between wettability, which ensures the supply of a thin layer of water, and localized heat to support evaporation is needed for efficient performance. A 5% laser power provided the optimal balance between heat absorption and wettability, resulting in an improved evaporation rate. This laser power was used to further investigate the effect of the scribing density.

### 3.5. Effect of Scribing Density (LPI)

**Figure 4:**
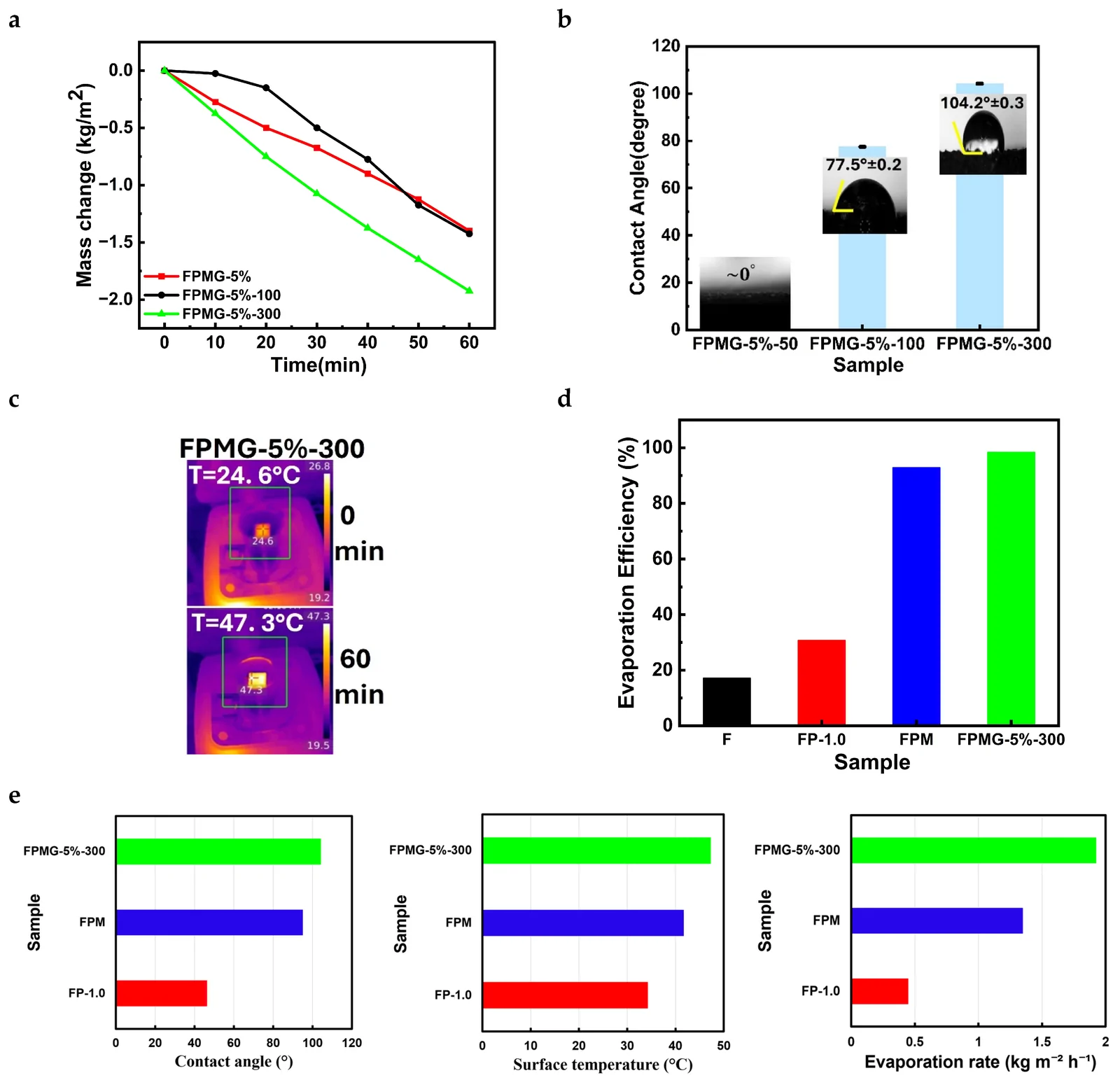
(**a**) The mass change in FPM5% samples under varying LPI values (50, 100, and 300). (**b**) Contact angle measurements of these samples at different LPI values. (**c**) IR images of the sample at 5% laser power and 300 LPI were taken at 0 min and after 60 min under one sun irradiation intensity. (**d**) Comparison of the energy efficiency of the best-performing samples under different conditions. (**e**) Contact angle, surface temperature, and their corresponding evaporation rates.

The effect of scribing density (LPI) on the evaporation rate was investigated using the FPMG-5% sample, where LPI denotes the number of laser-scribed lines per inch. This parameter directly influences surface morphology and photothermal performance. The tested LPI values were 50, 100, and 300, each affecting the material’s wettability and thermal properties differently. The results showed that the FPMG-5%-300 sample achieved the highest evaporation rate of 1.925 kg m^−2^ h^−1^, as shown in Figure 4a, indicating superior performance compared with the other tested configurations. The higher evaporation rate in the 300 LPI scribed sample can be attributed to changes in surface properties, as shown by significant variations in contact angle and surface temperature. At 50 LPI, the contact angle was 0°, suggesting a super-hydrophilic surface that facilitates water spreading. In contrast, at 300 LPI, the contact angle increased to 104.2° (Figure 4b), indicating reduced wettability. Despite this change, the 300 LPI scribing sample exhibited the highest surface temperature, reaching 47.3 °C Figure 4c, thereby enhancing thermal energy transfer and increasing the evaporation rate. Notably, the evaporation rate exceeded the theoretical limit of about 1.47 kg m^−2^ h^−1^. This is not unusual in porous materials as reported in the literature [21,22]. This is because the theoretical limit assumes evaporation enthalpy equals that of bulk water and that no energy comes from the environment. However, evaporation rates are higher in porous materials because the evaporation enthalpy decreases. Porous materials form intermediate or weakly bound water, which needs less energy to vaporize. Hence, such high evaporation rates are justifiable in porous and foam-like materials.

In this study, changes in contact angle, temperature, and evaporation rate during SSG fabrication were monitored. The FP-1.0 sample had the lowest values (~46.4°, ~34.3 °C, and 0.45 kg m^−2^ h^−1^, respectively), followed by the FPM sample (~95°, ~41.7 °C and 1.35 kg m^−2^ h^−1^, respectively). However, the FPMG-5%-300 sample has the highest values (~104.2°, ~47.3 °C and 1.925 kg m^−2^ h^−1^, respectively) as illustrated in Figure 4e. The contact angle for the FPMG-5%-300 sample is approximately twice that of the FP-1.0 sample. Moreover, the higher surface temperature of the last sample indicates effective heat localization, which plays an important role in improving SSG efficiency. Furthermore, light absorption and evaporation rate improved by approximately 4-fold compared with the FP-1.0 sample. Overall, to illustrate the development of the SSG system in this work, the evaporation efficiencies of the best-performing samples under different conditions (F, FP-1.0, FPM, and FPMG-5%-300) were calculated and compared, after accounting for the dark evaporation rates under the same ambient conditions, as shown in Figure 4d. The F sample exhibited the lowest efficiency, indicating its limited photothermal performance. In contrast, the FP-1.0 sample showed a significant improvement because the optimal PVA concentration enhances water interaction and light absorption. Incorporating MoS_2_ into sample FPM further improved evaporation efficiency by enhancing light-harvesting capabilities, reaching ~76%. The highest apparent evaporation efficiency of ~98% is observed in FPMG-5%-300 under a 5% laser power irradiation and 300 LPI scribing, demonstrating the synergistic effect of PVA, MoS_2_, and LIG in optimizing photothermal conversion and boosting evaporation rates. Clearly, in this work, integrating materials engineering with LIG enhanced the SSG system’s functionality. Hence, balancing surface hydrophilicity, which governs water supply to the evaporating interface, and surface temperature, which governs the energy available for vaporization, is essential for maximizing the evaporation rate (Figure 4e).

**Figure 5:**
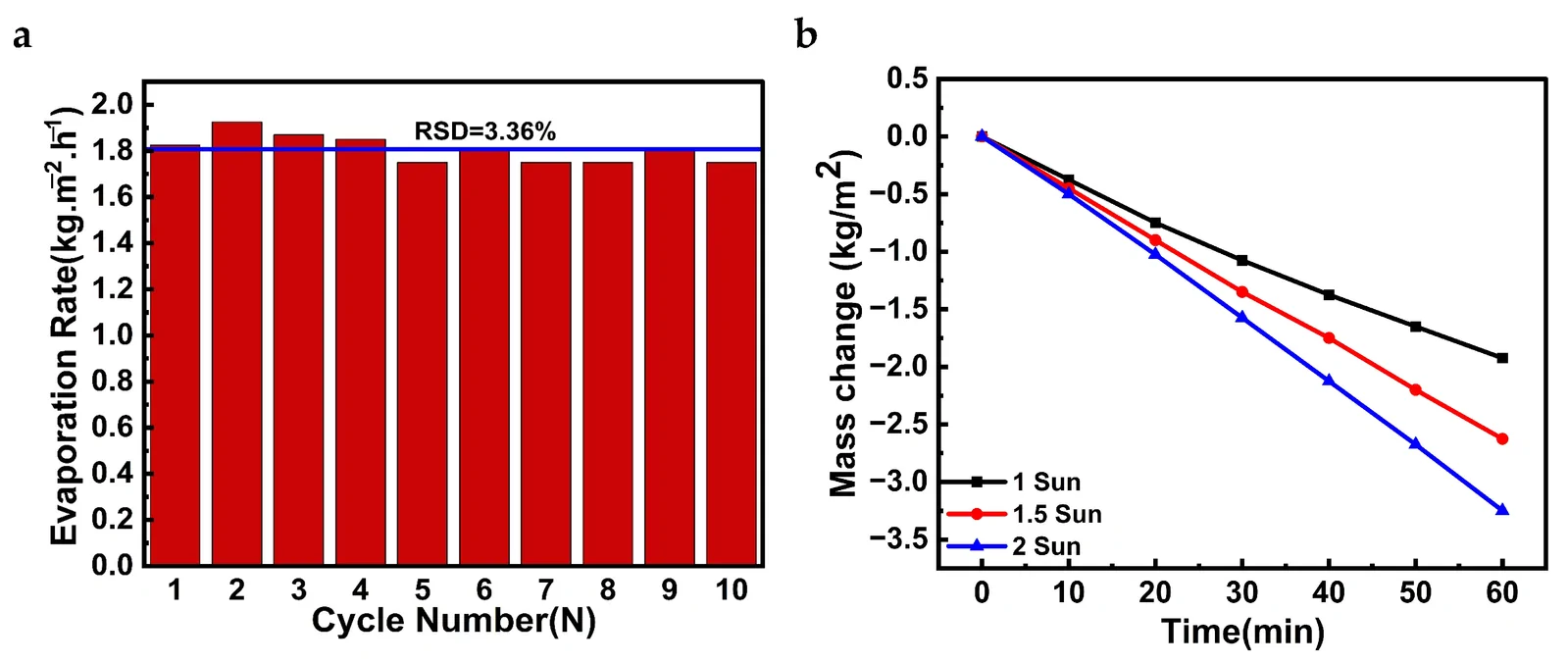
(**a**) The evaporation rate of the FPMG-5%-300 evaporator is over 10 cycles under 1 sun with RSD = 3.36%. (**b**) Illustrates the increase in mass change with increased solar intensity (1–2 sun) in a 3.5 wt% NaCl solution.

To evaluate the stability and robustness of the FPMG-5%-300 evaporator, solar evaporation experiments were conducted under 1 sun (1 kW/m^2^) for 10 cycles using a 3.5 wt% NaCl solution. After each cycle, the sample was removed and allowed to dry naturally before the next cycle began. As shown in Figure 5a, the relative standard deviation (RSD) of the FPMG-5%-300 evaporator was approximately 3.36%, indicating good precision (less than 5) and high reproducibility. The evaporation rate remained stable, and the evaporator’s morphology remained intact after 10 cycles. These results demonstrate the structural and performance stability of the FPMG-5%-300 evaporator, confirming its suitability for practical applications, even in saline environments.

Additionally, the evaporation performance of the FPMG-5%-300 evaporator was tested at different solar irradiation levels (1–2 sun) using the same 3.5 wt% NaCl solution. The evaporation rates, shown in Figure 5b, increase significantly with illumination intensity, from 1.925 kg m^−2^ h^−1^ (1 sun) to 3.25 kg m^−2^ h^−1^ (2 sun). These findings indicate that evaporation performance improves under higher solar irradiation.

### 3.6. Characterization of the SSG

#### Field Emission Scanning Electron Microscopy (FESEM)

**Figure 6:**
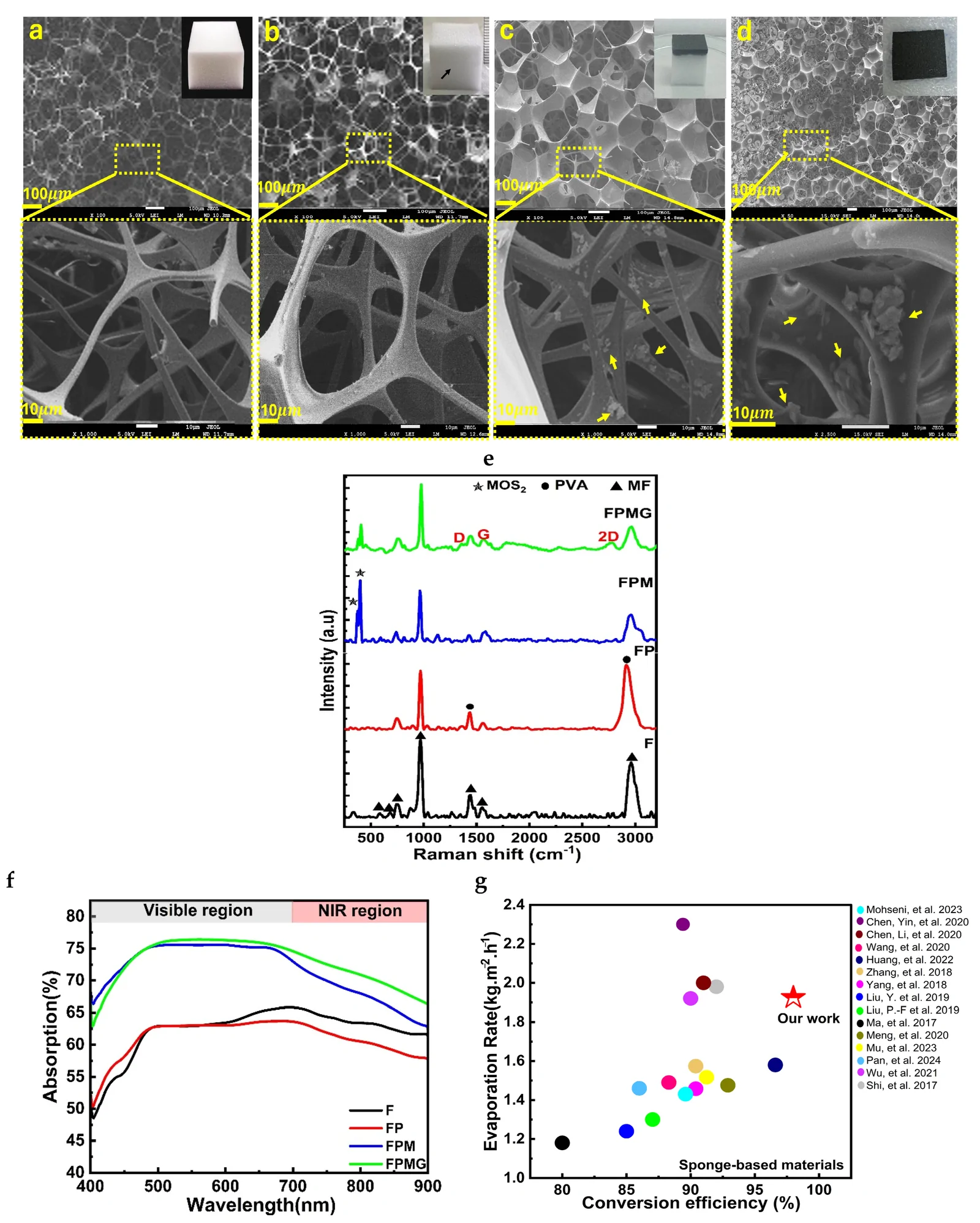
SEM images show a 3D network for the samples (**a**) F, (**b**) FP-1.0, (**c**) FPM and (**d**) FPMG at a scale of 100 μm, while the insets are the real sample photos; the black arrow indicates the PVA layer. Higher-magnification images at a 10 μm scale are also illustrated below, and the yellow arrows indicate the rough surface after MoS_2_ deposition. (**e**) The Raman spectra for F, FP-1.0, FPM and FPMG, where the D, G, and 2D bands confirm graphitization. (**f**) The absorption spectra of the fabricated evaporators, and (**g**) Performance comparison of evaporation rates in this work with previous references [12,21,22,24,28,29,30,31,32,33,34,35,36,37,38] for sponge-based materials.

FESEM was used to study the surface morphologies of pristine MF before and after modification. Images are shown at a scale of 100 μm, and for clarity, images at a 10 μm scale (high magnification) are also shown (Figure 6a–c). FESEM images (Figure 6a) reveal the characteristic porous structure of untreated MF, featuring high porosity. The untreated MF (F sample) exhibits a 3D network (or skeleton) with a smooth surface and several larger pores, making it suitable for solar seawater steam generators, as supported by previous studies [24]. MF tends to absorb water into its pores upon submersion, leading to saturation. However, its fine fibers and large pore spacing limit bonding sites and adhesion, reducing water resistance and evaporation efficiency. As shown in Figure 6b, PVA treatment modifies the MF structure (FP-1.0 sample) by reducing pore size and partially closing larger pores, forming a coating that creates either closed or open cells with labyrinth-like pathways, thereby increasing spatial density and internal stress. The rougher micro-surface of the (FP-10 sample) indicated successful PVA grafting. This structural improvement explains the higher evaporation rate achieved in the PVA-treated samples. Moreover, after MoS_2_ deposition, the surface morphology of the MoS_2_/PVA @MF (FPM sample) composite becomes rougher, with distinct protrusions uniformly distributed across the MF, as highlighted by yellow arrows in Figure 6c. Finally, LIG-modified FPM (FPMG sample) shows a significantly rougher surface morphology, as highlighted by yellow arrows in Figure 6d, thereby enhancing the surface hydrophobicity and thermal properties. These improved features and modifications did not disrupt the porous structure, which still maintains a 3D network, but rather increased the surface temperature and contact angle, thereby directly contributing to the higher evaporation rates observed and making the PVA-, MoS_2_-, and LIG-treated MF highly efficient for solar seawater evaporation applications.

### 3.7. Raman and Reflective Spectroscopies

The Raman spectra (Figure 6e) were recorded for the best-performing samples under different conditions (F, FP-1.0, FPM, and FPMG-5%-300) to investigate chemical changes and highlight the key vibrational peaks that characterized each sample. Characteristic Raman peaks of melamine-derived carbon foam are observed in the F sample. They appear at approximately 587 cm^−1^, 673 cm^−1^, 748 cm^−1^, 964 cm^−1^, 1444 cm^−1^, and 1563 cm^−1^, representing diverse triazine-ring vibrations [39], as well as at 2957 cm^−1^. The peak at 673 cm^−1^ corresponds to the ring-breathing II mode of carbon and involves an in-plane deformation of the triazine ring, while the ring-breathing I mode of the triazine ring of MF was observed at 964 cm^−1^. The peak at 1444 cm^−1^ is the envelope of three vibrations of the triazine ring and represents the A_1_ vibration; however, the vibration of species E’ was seen at both 1563 cm^−1^ and 587 cm^−1^ [28,29]. The peak at 748 cm^−1^ corresponds to trigonal ring breathing modes [40]. The strong peak at around 2957 cm^−1^ corresponds to the C–H stretching band, since the MF is made of Melamine Formaldehyde [41,42]. Additionally, the FP-1.0 spectrum shows representative PVA peaks at around 1400 cm^−1^ and 2928 cm^−1^ assigned to the CH_2_ antisymmetric deformation (shear mode) and the valence C–H symmetric stretching vibration of CH_2_, respectively [43,44]. The Raman spectrum of the FPM sample shows two sharp vibrational peaks at around 380 cm^−1^ and 404 cm^−1^ assigned to the in-plane $E_{2g}^{1}$ and out-of-plane A_1g_ modes of a typical lamellar MoS_2_ compound, respectively [12]. In the FPMG spectrum, distinct peaks are attributed to MF, PVA, and MoS_2_ materials. Moreover, three additional peaks are observed in the FPMG sample, namely the D, G, and 2D bands, at approximately 1350, 1565, and 2700, indicating that sp^2^ -bonded graphitic carbon was formed after the laser treatment. The D band arises from the breathing mode of sp^2^ six-membered carbon rings activated by defects and disorder; the G band indicates in-plane stretching of the C-C bond in the graphitic structure, and the 2D band represents a second-order overtone of the D-type phonon, which is sensitive to the order and stacking of the layers [45]. The peak intensity ratios I_D_/I_G_ and I_2D_/I_G_ were about 0.77 and 0.63, respectively. The I_D_/I_G_ indicates a moderate defect density, and an I_2D_/I_G_ ratio below 1 is consistent with a multilayer, defective carbon structure. Because the layer-number criteria established for pristine graphene do not directly apply to turbostratic LIG, the number of layers cannot be determined from Raman alone; hence, we describe the material as a porous, multilayer, defective graphitic (graphene-like) carbon [14]. Therefore, it can be concluded that the MF, PVA, MOS_2_, and laser-induced graphene are successfully incorporated into the produced FPMG sample.

In another aspect, to study the optical properties, the Vis-NIR absorption spectra of the F, FP-1.0, FPM, and FPMG-5%-300 samples were measured over the 400–900 nm range, as shown in Figure 6f. The absorption (A) can be calculated from the measured transmitted (T) and reflected (R) light using the formula A = 1−T−R, where T is very low [24]. The F sample exhibited relatively low absorption, starting at approximately 50% and peaking at ~65%. With the addition of 1 wt% PVA to sample FP-1.0, the absorption decreased, reaching a maximum of ~63%; this might be due to increased reflection. Incorporating MoS_2_ into sample FPM further enhanced absorption, peaking at ~75%. Sample FPMG, containing LIG, exhibited the highest absorption across the spectrum, peaking at ~77%. Although the peak absorption difference between FPM and FPMG is minimal, FPMG performs better, particularly in the NIR region (700–900 nm), where it maintains higher absorption. In contrast, the FPM sample shows a noticeable decline. This significant improvement highlights the synergistic effect of PVA, MoS_2_, and LIG in enhancing light-harvesting efficiency, making FPMG an ideal candidate for SSG.

Figure 6g demonstrates that our developed solar evaporation system achieves a competitive evaporation rate of 1.925 kg m^−2^ h^−1^, compared to many sponge-based materials [12,21,22,24,28,29,30,31,32,33,34,35,36,37,38]. Regarding the apparent evaporation efficiencies shown in Figure 6g, it should be noted that such efficiencies are calculated in the literature with different enthalpy values and different considerations for environmental energy inputs. No uniform standard protocol exists for measuring evaporation efficiencies of interfacial solar evaporators. Therefore, the reported evaporation efficiencies should be read as indicative rather than strictly comparable. However, we can conclude that the enhanced performance of the SSG fabricated in this work is attributed to the synergistic integration of LIG and MoS_2_, which enhances light absorption, heat transfer, and overall evaporation efficiency, making it an effective and sustainable solution for water desalination.

## 4. Conclusions

This study developed a highly efficient solar evaporation system using an MF coated with PVA and MoS_2_ layers, and a modified surface treated with optimized laser parameters, including laser power and scribing line density (LPI), to enhance thermal performance. The optimal PVA concentration of 1 wt% improved surface wettability, balancing water spreading and heat transfer, while MoS_2_ enhanced light absorption due to its excellent multi-band absorption properties. By incorporating LIG, an optimal sample (FPMG-5%-300), combining 1 wt% PVA and MoS_2_ with laser irradiation, achieved superior performance through surface treatment at 300 LPI and 0.5 W laser power. This increased light absorption from about 65% in the baseline sample (F) to 77% in the optimal sample. This increase significantly improved light-to-heat conversion, achieving an evaporation rate of 1.925 kg m^−2^ h^−1^ in saline water (3.5% NaCl) under a solar irradiance of 1 kWm^−2^, which increased to 3.25 kg m^−2^ h^−1^ when the irradiance was doubled. The optimal sample also demonstrated excellent operational stability over 10 consecutive cycles, with an RSD of 3.36%, maintaining consistent performance without significant surface degradation and exhibiting high reproducibility and precision. This combination of high efficiency, low cost, and simplicity makes the developed system a sustainable and scalable solution for solar-driven water desalination.

## Data Availability

The original contributions presented in this study are included in the article/Supplementary Material. Further inquiries can be directed to the corresponding author.

## Supplementary Materials

The following supporting information can be downloaded at: https://www.mdpi.com/article/10.3390/polym18212778/s1, Figure S1: Schematic illustration of the fabrication process for (a) FP, and (b) FPM samples. (c) A schematic representation of the experimental setup for SSG measurements, using an irradiation intensity of one sun and 3.5% saline water, created in BioRender. Ganash, E. (2026) https://BioRender.com/hpflk8d; Note S1: Evaporation Efficiency Calculation for FPMG-5%-300 sample.
