# Supplementary material for: Fabrication of Laser-Induced Graphene/MoS2/PVA@Melamine Evaporators for Rapid Solar Steam Generation
Source: Polymers (Basel). 2026 Nov 7;18(21):2778. doi: 10.3390/polym18212778 (PMC13766198; doi:10.3390/polym18212778)
Supplement: Supplementary file 1 [file polymers-18-02778-s001.zip › polymers-4495207-supplementary.pdf]

Supplementary Information

Fabrication of Laser-Induced Graphene/MoS_2/PVA@Melamine Evaporators for Rapid Solar Steam Generation

Entesar A. Ganash *, Salihah A. Al-zahrani, Hala A. Al-Jawhari and Reem M. Altuwirqi

Physics Department, Faculty of Science, King Abdulaziz University, Jeddah 21589, Saudi Arabia; salihahalzahrani1@gmail.com (S.A.A.-z.); hala.aljawhari@gmail.com (H.A.A.-J.); raltuwirqi@kau.edu.sa (R.M.A.)
* Correspondence: eganash@kau.edu.sa

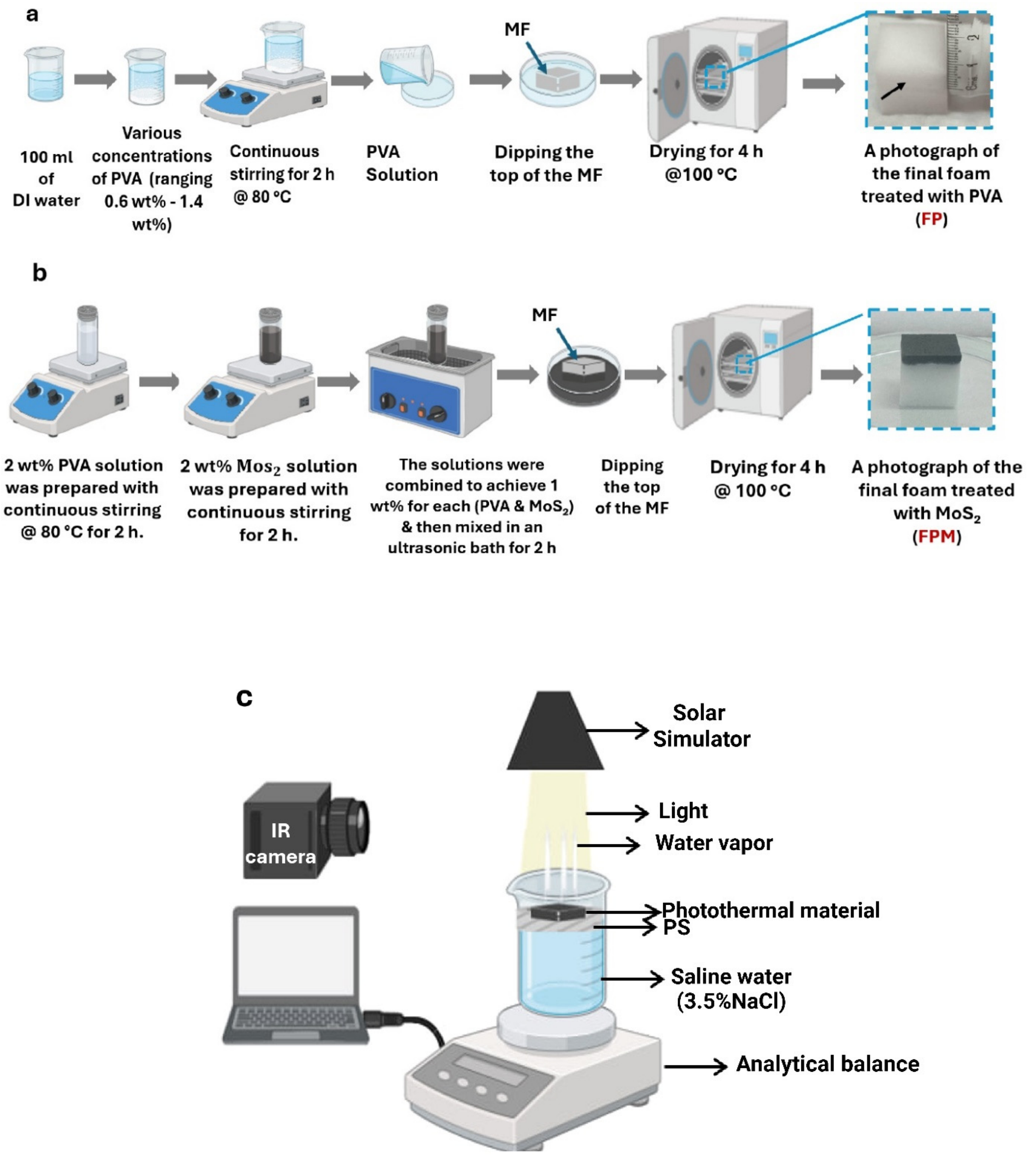

Figure S1: Schematic illustration of the fabrication process for **(a)** FP, and **(b)** FPM samples. **(c)** A schematic representation of the experimental setup for SSG measurements, using an irradiation intensity of one sun and 3.5% saline water, created in BioRender. Ganash, E. (2026) https://BioRender.com/hpflk8d

Note S1: Evaporation Efficiency Calculation for FPMG-5%-300 sample

The rate of water evaporation (v) is given by:

$$v = \frac{\Delta m}{S \times t} \qquad \text{(S1)}$$

Where

Δm: change in mass during water evaporation (kg),

S: area of the solar absorber (m^2),

T: irradiation time (h).

The water evaporation efficiency (η) due to solar radiation, taking into account the dark evaporation rate v_{dark}, is:

$$\eta = \frac{(v_{light} - v_{dark})\, h_{LV}}{C_{opt}\, q_{in}} \qquad \text{(S2)}$$

v_{light} = 1.925 kg m^{-2} h^{-1}

v_{dark} = 0.5 kg m^{-2} h^{-1}

h_{LV} is the total enthalpy

q_{in} = 1 kW/m^2,

C_{opt} = 1 for no concentrator

The total enthalpy is the addition of the sensible heat and the latent heat of vaporization:

$$h_{LV} = c(T - T_0) + h_v$$

The specific heat capacity of seawater (at standard salinity of 35 g/kg and room temperature): c = 4.0 kJ kg^{-1} K^{-1}

The membrane temperature, T = 47.3 ^oC

The initial water temperature, T_0 = 23 ^oC

h_v = 2389.2 kJ/kg at T = 47.3 ^oC (From steam tables of the National Institute of Standards and Technology (NIST).

$$h_{LV} = c(T - T_0) + h_v = 97.2\frac{kJ}{kg} + 2389.2\frac{kJ}{kg} = 2486.4\frac{kL}{kg}$$

$$\eta = \frac{(v_{light} - v_{dark})\, h_{LV}}{C_{opt}\, q_{in}} = \frac{(1.925 \text{ kg } m^{-2}\, h^{-1} - 0.5 \text{ kg } m^{-2}\, h^{-1})(2486.4\,\frac{kJ}{kg})}{(1)\left(1\,\frac{kW}{m^2}\right)(3600\frac{s}{h})} = 0.9842 = 98.42\%$$
